# Exophytic lesion in the anterior maxilla

**DOI:** 10.1002/ccr3.8727

**Published:** 2024-03-27

**Authors:** Ryan McConville, Amanda Mary Willis

**Affiliations:** ^1^ Queen's University Belfast Dentistry Belfast UK

## Abstract

A high index of suspicion is required for any rapidly expanding lesion in the oral cavity especially when associated with mobility of the dentition.

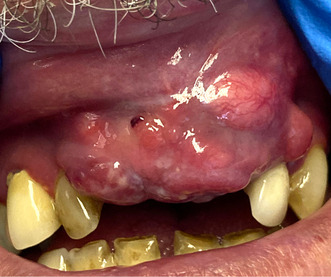

## 1 CASE REPORT

A 65‐year‐old man was referred to the Oral Medicine Department by his General Dental Practitioner for investigation of swelling in the anterior maxilla, which had been present for 6 weeks and associated with mobility in the upper central incisors. There was no resolution of the lesion following extraction of the central incisors and three courses of amoxicillin, which were prescribed by the GDP who initially believed the lesion was of an infective etiology.

The patient's medical history was significant for HIV which was diagnosed approximately 20 years ago and is managed with a combination antiretroviral and Co‐trimoxazole. The patient's viral load was undetectable. He was a never smoker and consumed 10 units of alcohol per week.

**FIGURE 1:**
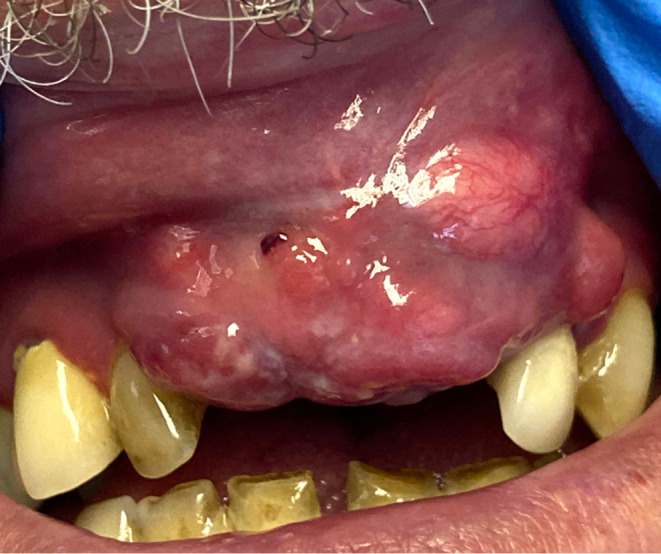
Exophytic lesion upper anterior maxilla and upper labial mucosa.

Extra orally, there was no lymphadenopathy. On intra oral examination a firm, nodular, exophytic soft tissue swelling was noted in the upper anterior maxilla extending to involve the labial sulcus (Figure 1).

Clinical photographs were carried out by the medical imaging department, Royal Victoria Hospital, Belfast.

Based on the patient's history and physical examination, which one of the following is the most suspicious diagnosis?

- Pyogenic granuloma
- Peripheral giant cell granuloma
- Plasmablastic lymphoma
- Kaposi Sarcoma

## 2 DIAGNOSIS

**FIGURE 2:**
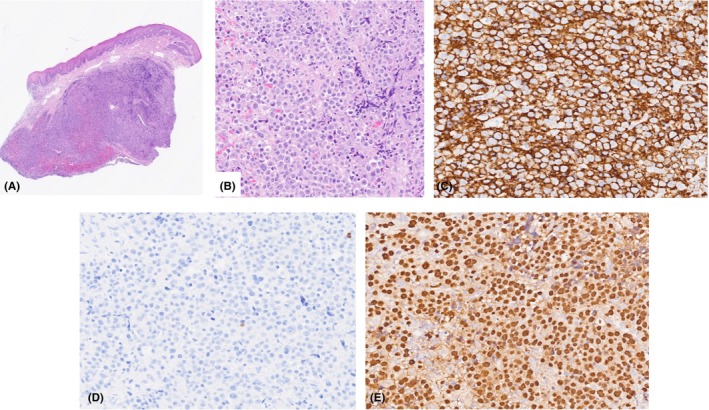
(A, B) Biopsy sections stained with H&E. (C) Immunohistochemistry positive for CD 138‐ a plasma cell marker. (D) Negative immunohistochemistry for CD 20—a B‐cell marker. (E) EBER ISH—Epstein Barr virus encoded RNA in‐situ hybridisation positive.

The correct diagnosis is C, Plasmablastic lymphoma (PBL). An incisional biopsy with a size 15 scalpel was undertaken and demonstrated extensive infiltration of the lamina propria with sheets of neoplastic plasmablasts (Figure 2 A,B). Immunohistochemistry was positive for CD 138 and MUM 1 which are plasma cell markers and negative for CD 20, a mature B cell marker (Figure 2C,D). There was also marked positivity for Epstein–Barr virus‐encoded RNA in the sample (Figure 2E). These investigations confirmed the diagnosis as Epstein Barr Virus‐positive PBL, consistent with viral‐induced immunosuppression.

PBL is a rare but aggressive subtype of diffuse large B‐cell lymphoma (Non‐Hodgkin lymphoma). It is estimated that PBL comprises 2% of HIV‐related lymphoma cases. It has a male predominance (3:1), with a median age of diagnosis in HIV‐positive patients of 42 years.^1^ It is strongly associated with immunodeficiency with 79% of cases having a concomitant HIV infection and 75% having an associated EBV infection.^2^ The Plasmablast is the precursor of the plasma cell and it is proposed that EBV leads to the prevention of apoptosis of these cells.^3^

The most notable feature of PBL is its predilection for the oral cavity with 66% cases presenting here.^2^ It most commonly presents as an expanding mass lesion on the gingiva or palate. Patients can also present with B symptoms: fevers, weight loss and night sweats in up to 40% of cases, however lymph node involvement is less frequently seen in these patients.^4^ Currently available chemotherapy fails to achieve good results and the prognosis of patients with PBL is generally poor with a median overall survival of 5–15 months.^3^

## 3 OUTCOME

The patient was referred to Hematology where he had a PET‐CT scan which showed subtle uptake in the maxilla and no further uptake seen elsewhere. He is currently undergoing chemotherapy and consolidation radiotherapy to his anterior maxilla under the care of the hematology team.

## AUTHOR CONTRIBUTIONS

**Ryan McConville:** Methodology; writing – original draft. **Amanda Mary Willis:** Conceptualization; supervision; writing – review and editing.

## CONFLICT OF INTEREST STATEMENT

All authors have no conflicts of interest to disclose.

## FUNDING INFORMATION STATEMENT

Funding from Queen's University Belfast.

## CONSENT

Written informed consent was obtained from the patient to publish this report in accordance with the journal's patient consent policy.

## Data Availability

The patients' data are not publicly available on legal or ethical grounds.
